# Normalisation of surfactant protein -A and -B expression in the lungs of low birth weight lambs by 21 days old

**DOI:** 10.1371/journal.pone.0181185

**Published:** 2017-09-26

**Authors:** Jia Yin Soo, Sandra Orgeig, Erin Victoria McGillick, Song Zhang, I Caroline McMillen, Janna L. Morrison

**Affiliations:** 1 Early Origins of Adult Health Research Group, School of Pharmacy & Medical Sciences, Sansom Institute for Health Research, University of South Australia, Adelaide, SA, Australia; 2 Molecular & Evolutionary Physiology of the Lung Laboratory, School of Pharmacy & Medical Sciences, Sansom Institute for Health Research, University of South Australia, Adelaide, SA, Australia; University of Giessen Lung Center, GERMANY

## Abstract

Intrauterine growth restriction (IUGR) induced by placental restriction (PR) in the sheep negatively impacts lung and pulmonary surfactant development during fetal life. Using a sheep model of low birth weight (LBW), we found that there was an increase in mRNA expression of surfactant protein (*SP)-A*, *-B* and *-C* in the lung of LBW lambs but no difference in the protein expression of SP-A or -B. LBW also resulted in increased lysosome-associated membrane glycoprotein *(LAMP)-3* mRNA expression, which may indicate an increase in either the density of type II Alveolar epithelial cells (AEC) or maturity of type II AECs. Although there was an increase in glucocorticoid receptor (*GR*) and 11β-hydroxysteroid dehydrogenase (*11βHSD*)*-1* mRNA expression in the lung of LBW lambs, we found no change in the protein expression of these factors, suggesting that the increase in *SP* mRNA expression is not mediated by increased GC signalling in the lung. The increase in *SP* mRNA expression may, in part, be mediated by persistent alterations in hypoxia signalling as there was an increase in lung *HIF-2α* mRNA expression in the LBW lamb. The changes in the hypoxia signalling pathway that persist within the lung after birth may be involved in maintaining SP production in the LBW lamb.

## Introduction

Intrauterine lung development and maturation are critically important for postnatal survival of the neonate. An important step in fetal lung development in late gestation is the maturation of the pulmonary surfactant system, which consists of a complex mixture of ∼90% lipids and 8–10% surfactant proteins (SP) [1]. Pulmonary surfactant is synthesised in type II alveolar epithelial cells (AEC), stored in lamellar bodies and under appropriate conditions is secreted via exocytosis into the alveolar space [2]. SP-B and -C, which are hydrophobic surfactant proteins, interact with the lipid component to facilitate adsorption of the surfactant film to the air-liquid interface of the alveoli. This film reduces the work of breathing by maintaining stability between alveolar units and preventing adherence of the alveolar walls during expiration [3]. SP-A and -D play important roles as effectors of the pulmonary innate immune system [4,5].

SP expression by type II AECs is under the control of a range of factors, including glucocorticoids (GC) [6,7], hypoxia [8] and peroxisome proliferator-activated receptor γ (PPARγ) [9]. GC increase the mRNA expression of SP-A, -B and -C indirectly through transcription factors such as thyroid transcription factor-1 (TTF-1) and cofactors such as GATA binding protein 6 (GATA-6) [10,11]. Hypoxia plays an important role because SP expression in the lung is reduced in hypoxia inducible factor-2α (HIF-2α) knockout mice, which results in respiratory distress syndrome [12,13]. Other transcription factors also play a role because administration of rosiglitazone in rodents, a PPARγ agonist, increases lung maturation by increasing SP expression [9].

Intrauterine growth restriction (IUGR), defined as a birth weight less than the 10^th^ centile for gestational age, affects more than 20 million newborns globally, and is particularly prevalent in developing countries [14,15]. Placental insufficiency is the most common cause of IUGR, which reduces both oxygen and nutrient transfer to the fetus [16,17]. There is a growing body of evidence that IUGR negatively impacts the growth and maturation of the fetal lung, with a decreased functional capacity persisting into later life [18,19]. Respiratory function is compromised in IUGR lambs as evidenced by lower functional residual capacity and total lung capacity compared with Control lambs at 8 weeks after birth [20]. IUGR alters lung structure with increased collagen deposition in rats [21] and reduced gas exchange surface in the late gestation fetal sheep [22]. An accumulation of glycogen in type II AECs in a maternal undernutrition model resulting in IUGR in rats suggests a delay in type II AEC differentiation [23]. Different sheep models of late gestation onset IUGR resulted in either increase SP-B mRNA expression, that is [24] or is not [13,25] associated with increased plasma cortisol concentration, or no change in SP mRNA expression [26] in late gestation. Late gestation onset IUGR also resulted in SP-B mRNA expression increase in 8 week old lambs [20]. In contrast, early gestation onset IUGR induced by PR is associated with delayed pulmonary surfactant maturation during late gestation, with a reduction in both surfactant protein mRNA and protein expression at 130 and 140 days (d) of gestation [27,28]. These changes are also accompanied by an increase in plasma cortisol concentrations [28] and there is evidence for a role of both hypoxia and glucocorticoid signalling [28]. In the present study, we aimed to determine whether early gestation onset IUGR would continue to supress SP expression in 21 day old lambs born with a low birth weight (LBW). Furthermore, we investigated potential molecular mechanisms, including GC and hypoxia signalling, cellular proliferation and PPARγ pathways, all of which regulate surfactant maturation in postnatal life.

## Materials and methods

All procedures were approved by the University of Adelaide Animal Ethics Committee and conformed to the NHMRC Australian code of practice for the care and use of animals for scientific purposes.

### Animals and surgery

Sixteen Merino ewes and their lambs were used in this study. Prior to mating, ewes were randomly divided into two treatment groups (Control and placental restriction (PR)). PR, leading to fetal growth restriction was induced in 6 ewes using carunclectomy [29–31]. General anaesthesia was induced in ewes with an intravenous injection of sodium thiopentone (1.25 g i.v., Pentothal, Rhone Merieux, Pinkenba, Queensland, Australia) and maintained with 2.5–4% halothane inhalation anaesthetic (Fluothane, ICI, Melbourne, Victoria, Australia) in oxygen. The uterus was incised and the majority of the caruncles, the sites of placentation, were removed from the uterus [27,29,32,33]. Antibiotics were administered intramuscularly to the ewe during surgery (153.5 mg procaine penicillin, 393 mg benzathine penicillin, 500 mg dihydrostreptomycin, Lyppards, South Australia, Australia). These ewes were allowed to recover for a period of at least 10–12 weeks before entering a mating program along with Control ewes [30].

#### Lamb birth and postnatal monitoring

Ewes gave birth spontaneously at term in individual pens after acclimatisation of at least two weeks [30]. The definition of average birth weight (ABW) and low birth weight (LBW) lambs was determined using a frequency distribution curve of birth weights of Control Merino singleton lambs from a separate cohort of 45 animals born from 1995–2004 under investigation by our laboratory. Birth weight of ABW lambs was 5.91±0.17 kg (n = 9, male = 6, female = 3) and that of LBW lambs was 3.78±0.17 kg (n = 7, male = 4, female = 3), which is 2 standard deviations below the average cohort mean. Information on the phenotype of the lambs and studies of cardiac growth and insulin signalling in skeletal muscles for this cohort have been published previously [30,34–38].

### Post mortem and tissue collection

Twenty-one days after birth, the lambs (ABW, n = 9; LBW, n = 7) were humanely killed with an overdose of sodium pentobarbitone injected into the jugular vein (Virbac Pty Ltd, Peakhurst, New South Wales, Australia). Lungs were dissected, weighed and samples were snap frozen in liquid nitrogen within 15 min and stored at -80°C for molecular analysis.

### Quantification of gene expression

#### Total RNA extraction

All essential information regarding our procedure is included as per the MIQE guidelines (7). Total RNA was extracted from ~50 mg lung samples (ABW, n = 9; LBW, n = 6) using Invitrogen Trizol Reagent Solution (Invitrogen Australia Pty. Ltd., Victoria, Australia) and RNeasy Mini Kit (QIAGEN, Victoria, Australia) according to the manufacturer’s instructions as previously described [39,40]. Total RNA was quantified by spectrophotometric measurements at 260 and 280nm in an Eppendorf BioPhotometer (Crown Scientific, NSW, Australia) and the 260/280nm ratio results were checked for protein and DNA contamination in each sample. Integrity of purified RNA was verified by assessment of the RNA bands run on a 1% agarose gel. cDNA was synthesised as previously described [39]. Controls containing either no Superscript III (No Amplification Control (NAC)) or no RNA transcript (No Template Control (NTC)) were used to test for genomic DNA and reagent contamination, respectively.

#### Quantitative real-time PCR

Based on the geNorm component of the qBase 2.0 relative quantification model (Biogazelle, Belgium), ribosomal protein P0 (*RpP0*, [30]), *β* actin (*ACTB*, [39]) and cyclophilin (*CYCLO*, [27]) were selected from a panel of eight candidate reference genes [41]. These reference genes were used as they were stably expressed across both groups (ABW vs. LBW) [42,43]. Primer pairs for plasminogen activator inhibitor (*PAI)*-1, DNA methyltransferase (*DNMT*) -1, -3a and -3b, fatty acid synthase (*FAS*) and sphingosine kinase (*SPHK)*-1 were designed (Table 1). Primers were validated to generate a single transcript as confirmed by melt-curve/dissociation curve and sequenced by the Australian Genome Research Facility Ltd. The mRNA expression of surfactant proteins (*SP-A*, *-B*, *-C* and *-D* [27,39]), GC signalling genes (glucocorticoid receptor (*GR*), mineralocorticoid receptor (*MR*) [39], 11β-hydroxysteroid dehydrogenase (*11βHSD*) -1 and -2 [39], GATA-binding protein (*GATA*) -6 [39], thyroid transcription factor (*TTF*) *-*1 [39]), hypoxia signalling and feedback (hypoxia inducible factor (*HIF*) -1α and -2α, prolyl hydroxylase domain (*PHD*) -1, -2 and -3) [44], hypoxia responsive genes (Vascular endothelial growth factor (*VEGF*) [44], jumanji demethylases1A (*JMJD1A*) [44], insulin-like growth factor (*IGF*) -2 [45], fms-related tyrosine kinase (*FLT*) -1 [44] and angiotensin converting enzyme (*ACE*) -1 [45]), peroxisome proliferator-activated receptors gamma (*PPARγ*) [46], PPARγ responsive genes (*SPHK-1* and *PAI-1*), genes involved in proliferation (proliferating cell nuclear antigen (*PCNA*, KI-67 *[28]*), genes regulating the cell cycle (*p27* and cyclin D1 (*CCND-1)* [47]), marker of lamellar bodies in type II AECs (lysosomal associated membrane protein (*LAMP*-3) [28]), surfactant lipid transporter (ATP-binding cassette, sub-family A, member 3 *(ABCA*3) [48]), markers of pulmonary surfactant lipid synthesis (phosphate cytidylyl transferase 1, choline, alpha (*PCYT1A*) [48] and *FAS*) and reference genes in lung samples were measured using Fast SYBR® Green Master Mix (Applied Biosystems, California, USA) on a ViiA7 Fast Real-time PCR system (Applied Biosystems, California, USA) as previously described [40]. The abundance of each transcript relative to the abundance of stable reference genes was calculated using DataAssist 3.0 analysis software (Applied Biosystems, California, USA) [43] and expressed as mRNA mean normalised expression (MNE) ± SEM [39,40].

**Table 1:** qRT-PCR primer sequences, concentrations and accession numbers for target genes that were designed and validated for this study.

Primer Name	Sequence 5’ → 3’	Primer Conc. (*μ*M)	Melting point (°C)	Size (bp)	Accession No.	Reference
***SP-A***			80	90	AF211856	[27]
*Forward*	AGCTCCAGGGCACACTCCATG	0.3				
*Reverse*	CTCCCACTTCCAGCATGGAC	0.3				
***SP-B***			84	153	AF107544	[27]
*Forward*	GGGCCCCACATTCTGGTGC	0.3				
*Reverse*	TCCTTGGCCATCTTGGTGAGG	0.3				
***SP-C***			84	149	AF076634	[27]
*Forward*	GCAAAGAGGTCTTGATGGAG	0.3				
*Reverse*	CAGGGCTCCTACGATCACC	0.3				
***SP-D***			76	62	AJ133002.1	[39]
*Forward*	GGCCACAGCCCAGAACAA	0.3				
*Reverse*	AAGTACCCTCCTTCCTGGTATCG	0.3				
***SPHK-1***			82	70	XM_002696204.2	
*Forward*	CCACTGCCCCCACCTGGTCTATGTG	0.45				
*Reverse*	CACACCCTTCCCATCCTTGG	0.45				
***PAI-1***			84	182	NM_001174114	
*Forward*	TCGACAGCAGATCCAAGAGGCAAT	0.45				
*Reverse*	TGAAGAAGTTGGGCATGAAACCGC	0.45				
***FAS***			81	95	AF479289.1	
*Forward*	CCCAGCTCAACGAAACCA	0.45				
*Reverse*	GACGAGGTCAACACCCTTCC	0.45				
***DNMT-1***			79	164	NM_001009473.1	
*Forward*	AGCAAGCGGAGACCAGAAGAGAAA	0.45				
*Reverse*	TCGGTCTTGGACACCACTGCTATT	0.45				
***DNMT-3a***			81	80	HQ202740.1	
*Forward*	TTTCCAATGTGCCATGACAGCGAC	0.45				
*Reverse*	GGCCCACTCGATCATCTGTTTGTT	0.45				
***DNMT-3b***			81	146	HQ202741.1	
*Forward*	CCAGTGGTTTGGTGATGGCAAGTT	0.45				
*Reverse*	TGGCTTTCTCCAGAGCATGGTACA	0.45				

Accession numbers refer to the published cDNA sequences from which the primer sequences were designed.

### Quantification of protein extraction

#### Protein extraction

∼50–100 mg of a subset of lung samples (ABW = 7, LBW = 6) were cut and sonicated (Kinematica PT-MR-3100, Lucerne, Switzerland) in 500 μL of homogenising buffer. Homogenates were centrifuged, the supernatant was recovered and the pellets were discarded.

#### Total protein quantification

The amount of protein in each extraction was determined using a micro Bicinchoninic acid (BCA) protein assay kit (PIERCE, Thermo Fisher Scientific Inc, Rockford, USA) with bovine serum albumin to generate a standard curve. The extracted protein was diluted with protein sample buffer in aliquots to a concentration of 5 mg/mL. Prior to Western blot analysis, samples were subjected to sodium dodecyl sulfate polyacrylamide gel electrophoresis (SDS-PAGE) and gels stained with Coomassie blue reagent (Thermo Fisher Scientific Inc., USA). This is a quality control step performed before Western Blotting to confirm equal loading of the proteins as performed previously [36,40,49–51].

#### Western blotting

Protein from each sample was subjected to SDS-PAGE as described above and transferred onto a nitrocellulose membrane using a wet transfer system [28,44]. The membranes were stained with 0.1% Ponceau S in 7% acetic acid to confirm proper transfer and washed with 7% acetic acid to remove the stain. The membranes were blocked in either 5% BSA in Tris-buffered saline with 1% Tween-20 (TBS-T; 10 mM Tris, 0.9% NaCl, pH 7.4, with 0.1% Tween 20) or 5% skim milk in TBS-T and then incubated with the respective primary antibody; goat anti-human SP-A antibody (Chemicon International, Billerica, MA) [28], mouse anti-human monoclonal antibody to SP-B (produced by Dr Y. Suzuki, Kyoto University, Japan and kindly donated by Prof Fred Possmayer, University of Western Ontario, Canada) [27], rabbit anti-human polyclonal antibody to 11βHSD-1 (Cayman Chemicals, MI, USA) [28], rabbit anti-human polyclonal antibody to 11βHSD-2 (Cayman Chemicals, MI, USA) [28], rabbit anti-mouse polyclonal antibody to GR (generously donated by Prof Vicki Clifton, Mater Medical Research Institute Limited, Australia) [28], goat anti-human polyclonal antibody GATA-6 (Santa Cruz Biotechnology, Inc, Texas, USA) [28], rabbit anti-human polyclonal antibody to TTF-1 (Santa Cruz Biotechnology, Inc, Texas, USA) [28], rabbit anti-human polyclonal antibody to PHD-1 (Novus Biologicals, CO, USA) [28,44] and rabbit anti-human polyclonal antibody to PHD-2 (Novus Biologicals, CO, USA) [28,44]. Membranes were then incubated with their respective horseradish peroxidase (HRP)-conjugated secondary IgG antibody (Cell Signalling Technology, Inc., Massachusetts, USA) and antigen-antibody complexes were detected by enhanced chemiluminescence using SuperSignal® West Pico chemiluminescent substrate (Thermo Fisher Scientific Inc., USA) using an ImageQuantTM LAS 4000 imager (GE Healthcare, Australia). Specific bands were quantified by densitometry using ImageQuant TL software (GE Healthcare, Australia) and data is expressed as arbitrary units (AU).

### Plasma cortisol radioimmunoassay

Total plasma cortisol concentration was measured using an ^125^I radioimmunoassay kit (GE Healthcare, Sydney, Australia) as previously described [27,52] in a blood sample collected from the lamb on the morning of the 20^th^ day after birth. The average efficiency of recovery of ^125^I cortisol using dichloromethane extraction was 90%. The sensitivity of the assay was 0.39 nmol/L. The rabbit anti-cortisol antibody cross-reacted 1% with cortisone and 17-hydroxyprogesterone and 0.01% with aldosterone, pregnenolone, estradiol, and progesterone. The inter- and intra-assay coefficients of variation (CV) were less than 10%.

### Lung tissue cortisol enzyme-linked immunosorbent assay (ELISA)

A subset of lung samples (~50–60 mg; ABW = 5, LBW = 6) were cut and sonicated (Kinematica PT-MR-3100, Lucerne, Switzerland) as previously described [53]. The samples were centrifuged at 1,500rcf (Beckman J6 Centrifuge), the supernatant removed from each sample and the pellets were discarded. Cortisol was extracted from the supernatant by combining supernatant with extraction buffer (Oxford Biomedical Research Inc., Michigan, USA) and ethyl ether (VWR, Qld, Australia) and vortexed for 1 min followed by 5 min phase separation. The organic phase was transferred into a glass tube and dried at 37°C with air for 30 min and re-suspended in extraction buffer.

Test samples and the cortisol standards were assayed in duplicate on a Cortisol Enzyme Immunoassay according to the manufacturer’s guidelines (EA65, Oxford Biomedical Research Inc., Michigan, USA) [54]. The plate was read on a standard 96-well colorimetric plate reader (450 nm; Victor^3^ Multilabel Plate Reader 1420, Perkin Elmer, Massachusetts, USA) and total cortisol concentration was determined from the standard curve. Results are expressed as total cortisol in lung tissue (pg/mg).

### Statistical analysis

All data are presented as mean ± standard error of the mean (SEM). Two-way ANOVA was performed to determine the effect of treatment (ABW vs LBW), the effect of sex (M vs F) and the interaction between the effects of treatment and sex on all parameters (Statistical Package for Social Sciences (SPSS) v17.0, Chicago, USA). In this cohort we saw no effect of sex and no interaction between the effects of treatment and sex for any parameter. Therefore, data for male and female animals were pooled and analysed using a Student’s unpaired t-test (ABW vs LBW). A probability level of 5% (*P*<0.05) was considered statistically significant.

## Results

### Effect of LBW on body and lung weight, plasma cortisol concentration and lung tissue cortisol in lambs at 21d after birth

LBW lambs had significantly decreased body weight both at birth and 21d after birth as previously published [30]. LBW lambs had a decrease in absolute lung weight (ABW, 206.86±10.47 g; LBW, 163.84±9.09 g; *P*<0.05). However, there was no change in relative lung weight (ABW, 15.68±0.64g/kg; LBW, 15.56±1.68 g/kg; *P*>0.05). There was also no significant difference in plasma cortisol concentration (ABW, 16,200 ± 4,900 pmol/L; LBW, 22,700 ± 5,600 pmol/L; *P*>0.05) or lung tissue cortisol concentration (ABW, 2.99 ± 0.60 pg/mg; LBW, 4.56 ± 1.0 pg/mg; *P*>0.05) on the day of post mortem between ABW and LBW lambs.

### Effect of LBW on SP mRNA and SP-A and -B protein expression in the lung of LBW lambs 21d after birth

**Fig 1:**
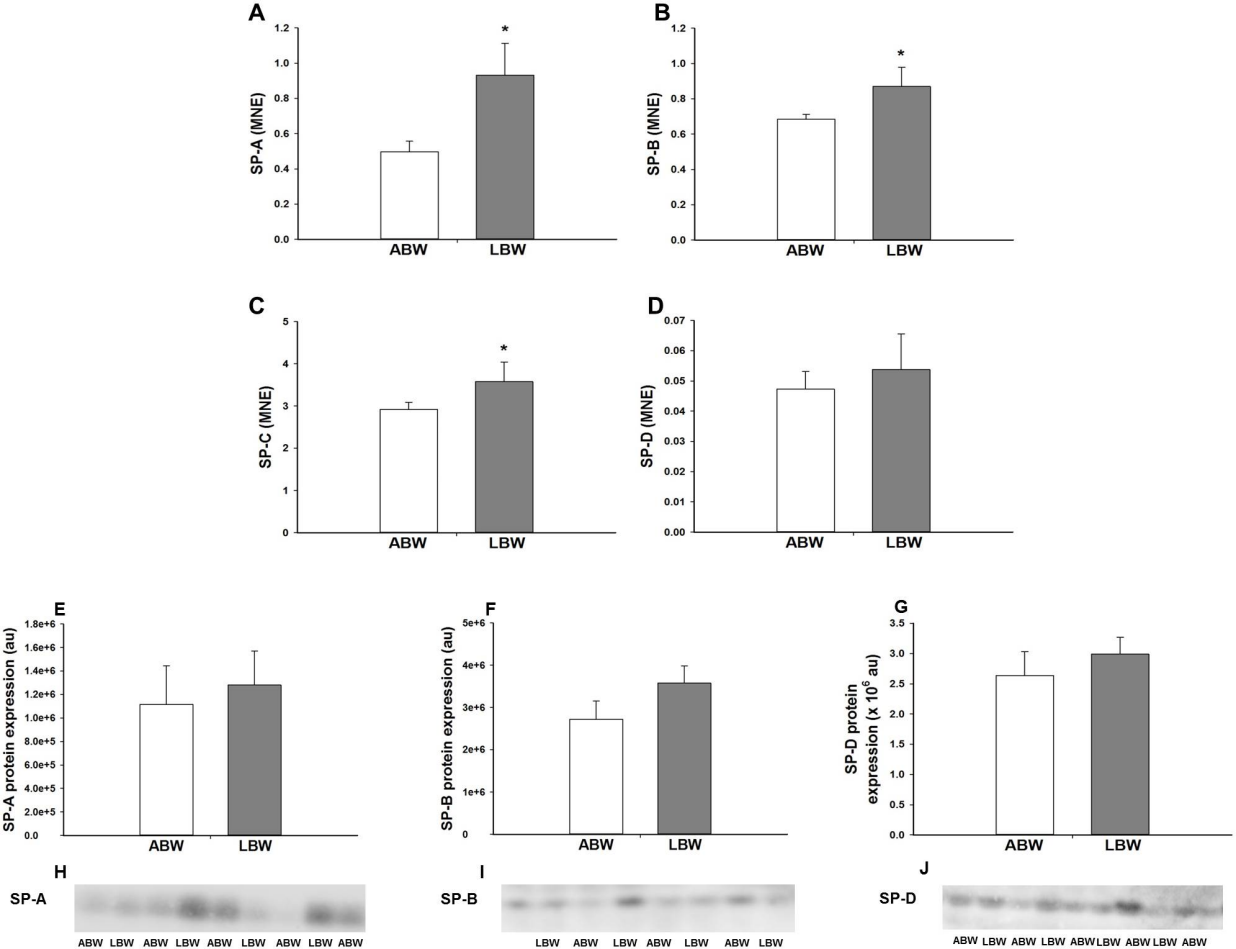
**There was higher mRNA expression of SP-A (A), -B (B) and -C (C), but not SP-D (D), in the lungs of the LBW lambs (n = 6; Closed bars) compared with ABW lambs (n = 9; Open bars). There was no difference in the protein expression of SP-A (E), -B (F) and SP-D (G) in the lungs of the LBW lambs (n = 6; Closed bars) compared with ABW lambs (n = 7; Open bars). Representative Western blot images from animals in each treatment group for SP-A (G),–B (H) and–D (J) are presented.** MNE, mean normalised expression; ABW, Average birth weight; LBW, Low birth weight; *P<0.05 from ABW.

There was higher *SP-A*, *-B* and *-C* mRNA expression in the lungs of LBW compared with ABW lambs, however, there was no difference in *SP-D* mRNA expression (Fig 1A, 1B, 1C and 1D). There was no difference in SP-A and -B protein expression (Fig 1E and 1F) in the lung between the ABW and LBW lambs. There was a significant negative correlation between birth weight and *SP-A* mRNA expression, but there was no significant relationship between birth weight and *SP-B*, *-C* or *-D* mRNA expression (Table 2).

**Table 2:** Regression analyses of *SP*, *GR*, *11βHSD* mRNA expression against birth weight and weight at 21 days and *SP* mRNA expression against plasma cortisol concentrations on postnatal day 20.

Variables	Equation	*N*	Adjusted *r*^2^	*P*
***SP mRNA expression (MNE) vs*. *Birth weight (kg)***				
*SP-A*	y = 1.84x – 0.23	14	0.565	**0.001**
*SP-B*		14	0.205	0.059
*SP-C*		14	0.108	0.135
*SP-D*		14	0.099	0.145
***GR*, *11βHSD mRNA expression (MNE) and plasma cortisol vs*. *Birth weight (kg)***				
*GR*	y = 0.19x – 0.015	14	0.489	**0.003**
*11βHSD-1*		13	0.191	0.076
*11βHSD-2*		13	0.016	0.292
Plasma cortisol concentration		9	0.413	0.062
Lung tissue cortisol concentration		11	-0.0120	0.372
***SP mRNA expression (MNE) vs*. *Weight at 21 days (kg)***				
*SP-A*	y = 2.09x – 0.012	15	0.356	**0.011**
*SP-B*		15	0.169	0.071
*SP-C*		15	0.049	0.212
*SP-D*		15	-0.026	0.436
***GR*, *11βHSD mRNA expression (MNE) and plasma cortisol vs*. *Weight at 21 days (kg)***				
*GR*	y = 0.228x – 0.001	14	0.582	**0.001**
*11βHSD-1*		13	0.092	0.292
*11βHSD-2*		14	0.051	0.217
Plasma cortisol concentration		10	0.079	0.219
Lung tissue cortisol concentration		11	-0.0976	0.747
***SP mRNA expression (MNE) vs plasma cortisol concentration***				
*SP-A*		10	0.051	0.259
*SP-B*		10	0.006	0.830
*SP-C*		10	0.156	0.259
*SP-D*		10	1.34*E*^-005^	0.992
***SP mRNA expression (MNE) vs lung tissue cortisol concentration***				
*SP-A*		10	0.146	0.150
*SP-B*		10	0.038	0.278
*SP-C*	y = 0.34x + 2.27	10	0.446	**0.021**
*SP-D*	y = 0.0092x + 0.026	10	0.4522	**0.020**

Regression equations are only provided in case of significant relationships. MNE, mean normalised expression.

### Effect of LBW on GC signalling and cofactors involved in GC signalling in the lung of lambs 21d after birth

**Fig 2:**
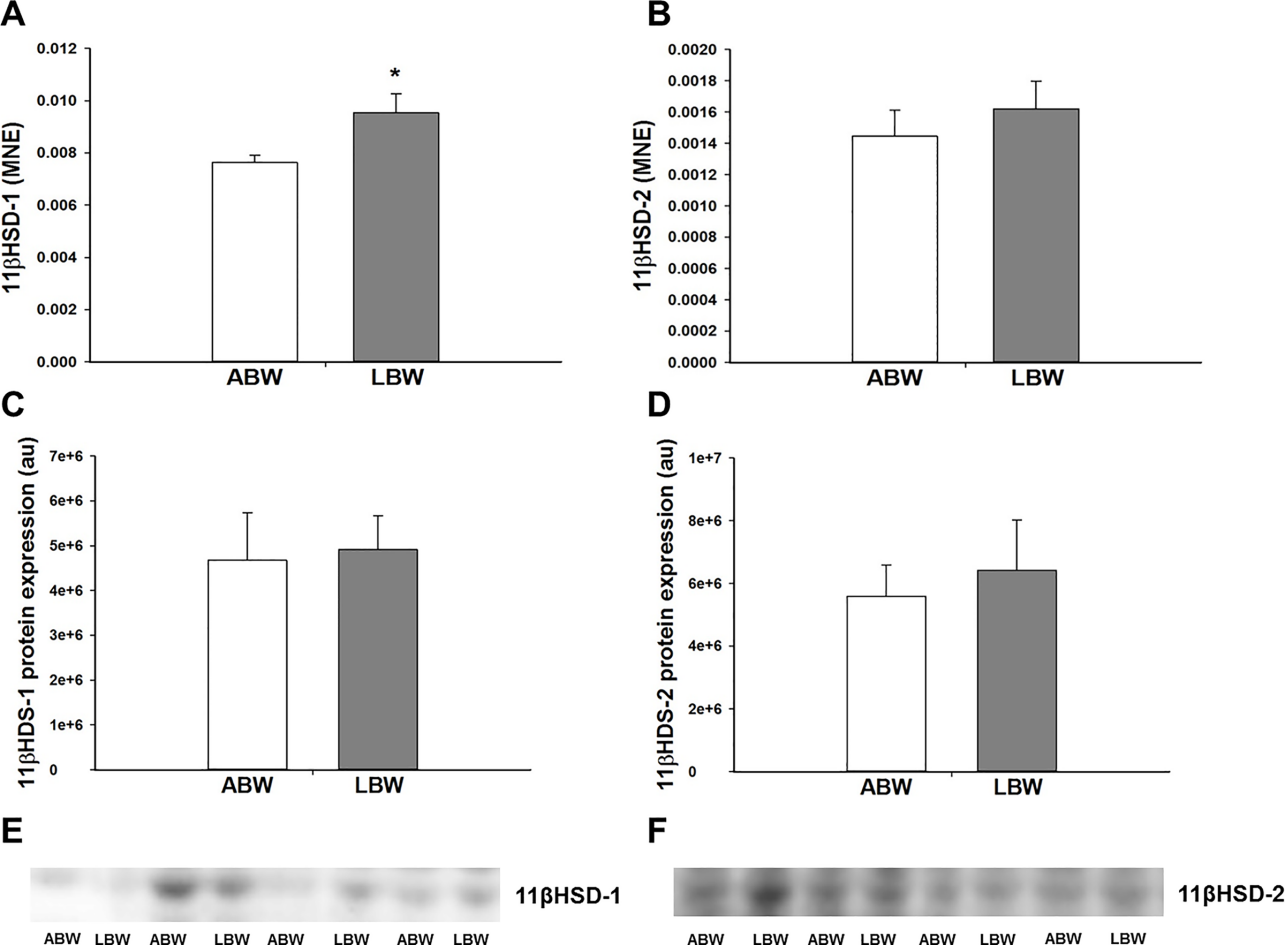
**There was higher mRNA expression of *11βHSD-1* (A) in the lungs of the LBW lambs (n = 6; Closed bars) compared with the ABW lambs (n = 8; Open bars). There was no difference in the mRNA expression of *11βHSD-2* (B) in the lungs of the LBW lambs (n = 6; Closed bars) compared with the ABW lambs (n = 8; Open bars). There was no difference in the protein expression of 11βHSD-1 (C) and 11βHSD-2 (D) between the lungs of the LBW lambs (n = 6; Closed bars) and ABW lambs (n = 7; Open bars). Representative Western blot images from animals in each treatment group for 11βHSD-1 (E) and 11βHSD-2 (F) are presented.** MNE, mean normalised expression; ABW, Average birth weight; LBW, Low birth weight; *P<0.05 from ABW.

**Fig 3:**
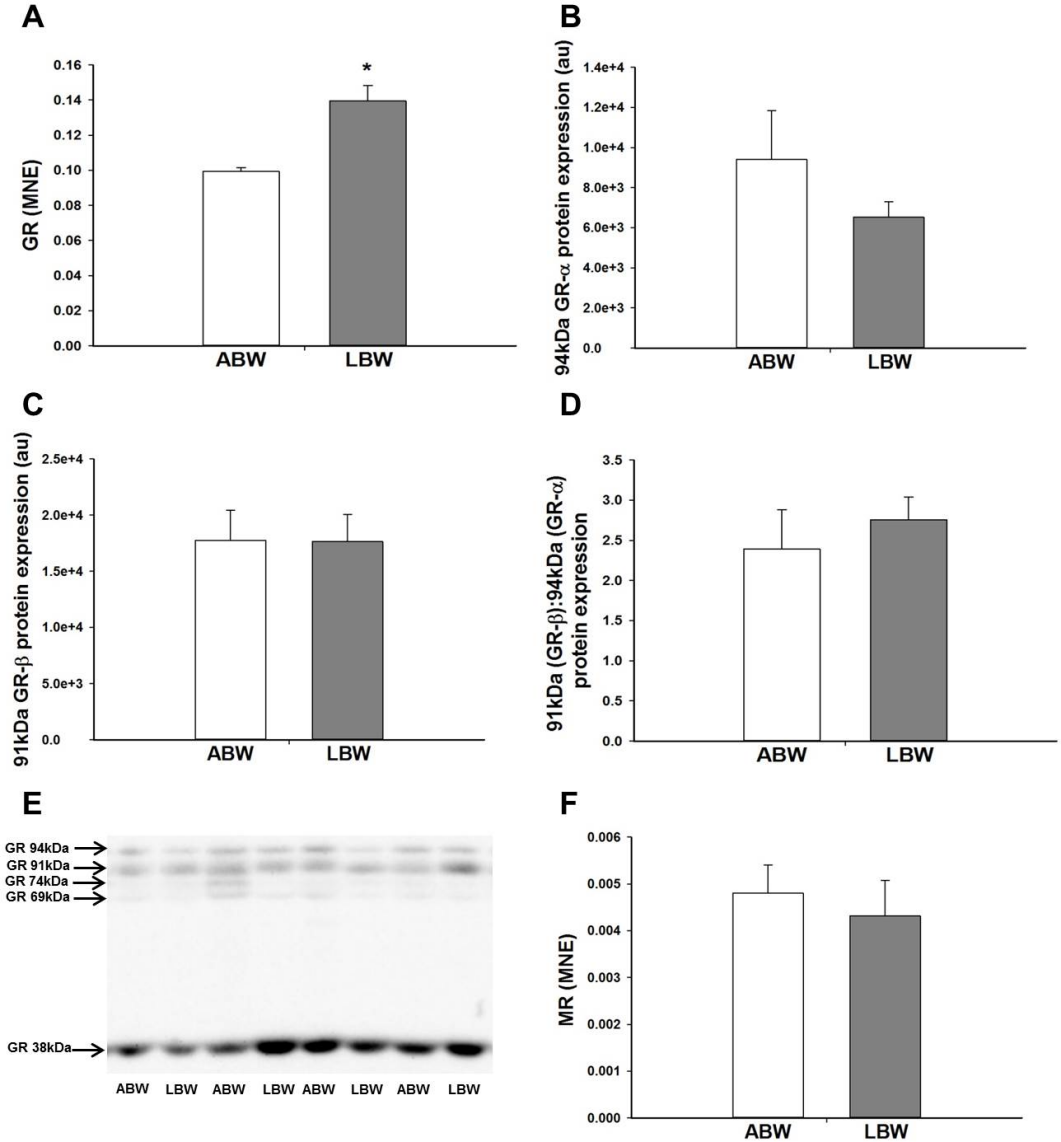
**There was higher mRNA expression of the *GR* (A) in the lungs of the LBW lambs (n = 7; Closed bars) compared with the ABW lambs (n = 6; Open bars). However, there was no difference in the protein expression of 94kDa GR-α (B), 91kDa GR-β (C) between the lungs of the LBW lambs (n = 6; Closed bars) and ABW lambs (n = 7; Open bars). There was no difference in the ratio of 91kDa GR-β to 94kDA GR-α protein expression (D) between the lungs of the LBW lambs (Closed bars) and ABW lambs (Open bars). A representative Western blot image from animals in each treatment group for GR (E) is presented. There was no difference in the mRNA expression of *MR* between the lungs of the LBW lambs (n = 6; Closed bars) and ABW lambs (n = 9; Open bars) (F).** MNE, mean normalised expression; ABW, Average birth weight; LBW, Low birth weight; *P<0.05 from ABW.

**Fig 4:**
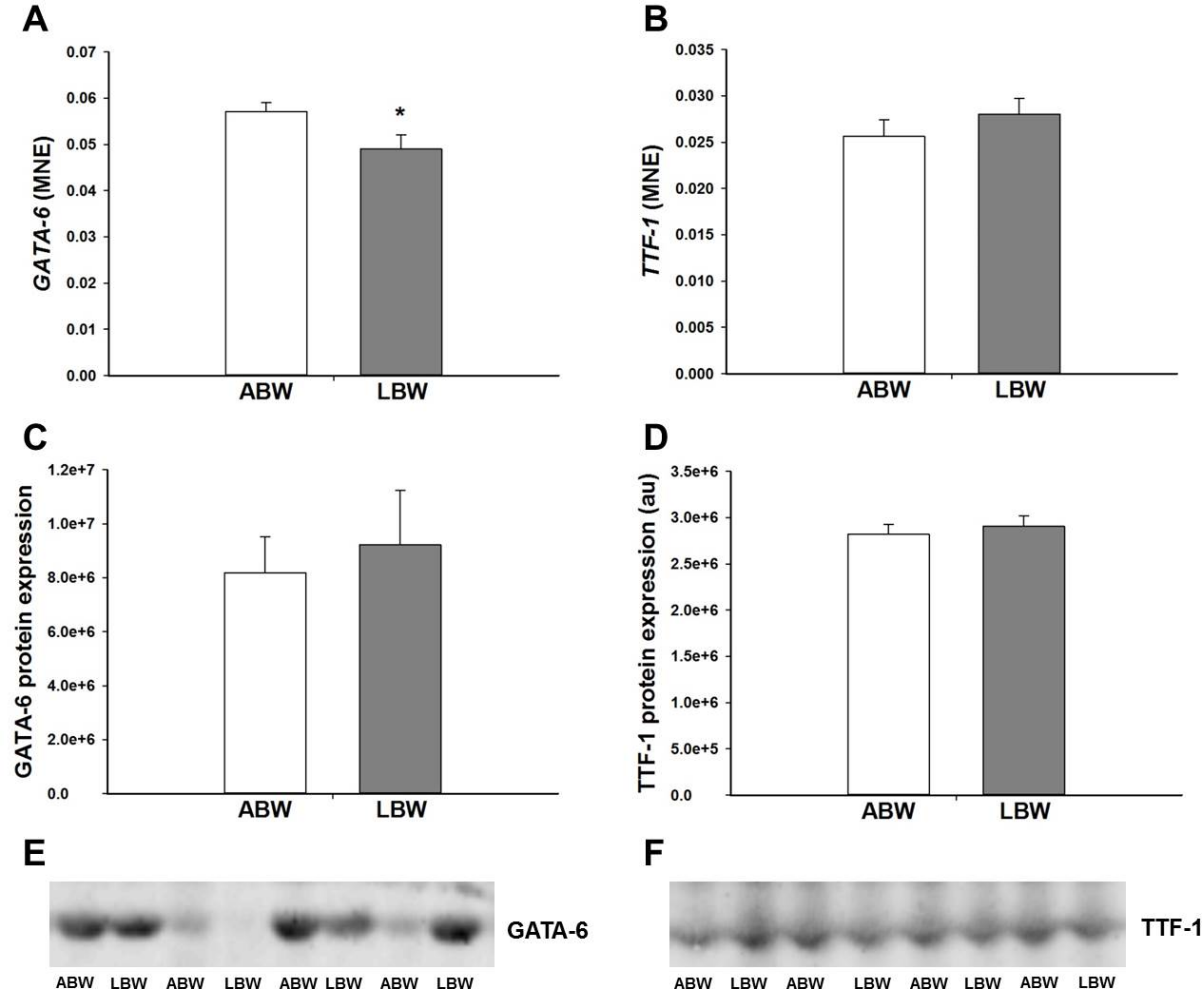
**There was lower mRNA expression of *GATA-6* (A) in the lungs of the LBW lambs (n = 6; Closed bars) compared with the ABW lambs (n = 9; Open bars). There was no difference in the mRNA expression of *TTF-1* (B) in the lungs of the LBW lambs (n = 5; Closed bars) compared with the ABW lambs (n = 8; Open bars). There was no difference in the protein expression of GATA-6 (C) and TTF-1(D) between the lungs of the LBW lambs (n = 6; Closed bars) and ABW lambs (n = 7; Open bars). Representative Western blot images from animals in each treatment group for GATA-6 (E) and TTF-1 (F) are presented.** MNE, mean normalised expression; ABW, Average birth weight; LBW, Low birth weight; *P<0.05 from ABW.

There was an increase in the mRNA expression of the GC activating isoform *11βHSD-1*, but not the GC deactivating isoform *11βHSD-2* in the lung of the LBW compared with the ABW lambs (Fig 2A and 2B). However, there was no difference in the protein expression of 11βHSD-1 and 11βHSD-2 between the lungs of ABW and LBW lambs (Fig 2C and 2D). LBW was associated with a significant increase in lung *GR* mRNA expression (Fig 3A) that resulted in a significant negative correlation between birth weight and *GR* mRNA expression (Table 2), but not in the lung protein abundance of GR. Furthermore, there was no difference in the protein expression of the 94kDa GR-α, 91kDa GR-β, 74kDa GR-P, 69kDA GR, 39kDA GR or ratio of GR-β to GR-α isoforms between LBW and ABW lambs (Fig 3B, 3C and 3D). There was also no difference in the mRNA expression of the *MR* (Fig 3F). LBW was also associated with a decrease in GC signalling cofactor *GATA-6* mRNA expression in the lungs (Fig 4A). However, there was no difference in the protein expression of GATA-6 in the lungs of the LBW and the ABW lambs (Fig 4C). There was no difference in either mRNA or protein expression of TTF-1 (Fig 4B and 4D).

### Effect of LBW on hypoxia signalling and feedback in the lungs of lambs 21d after birth

**Fig 5:**
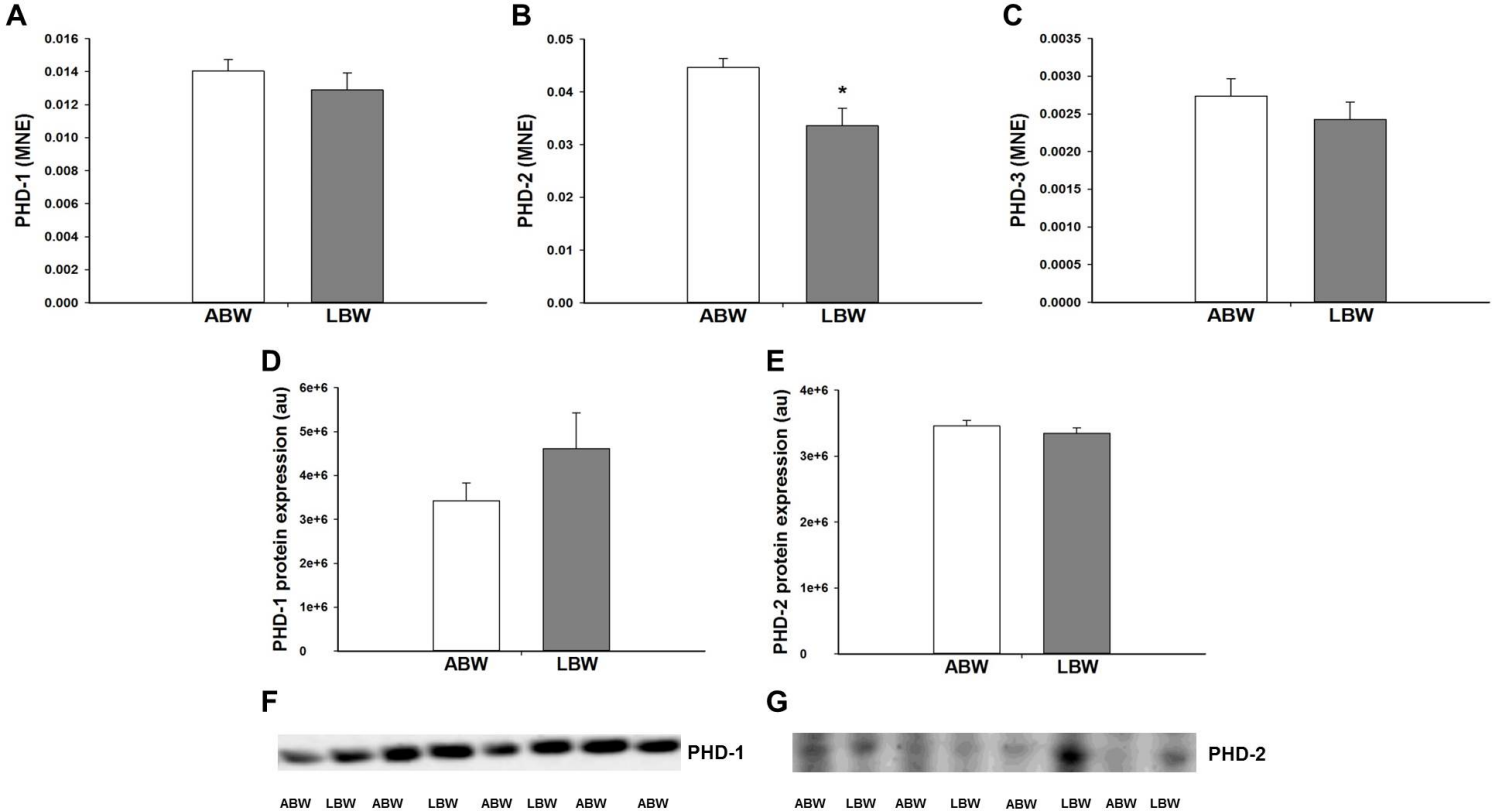
**There was no difference in the mRNA expression of *PHD-1* (A) and *PHD-3* (C) in the lungs of the LBW lambs (n = 6; Closed bars) compared with the ABW lambs (n = 9; Open bars). There was lower mRNA expression of *PHD-2* (B) in the lungs of the LBW lambs (n = 6; Closed bars) compared with the ABW lambs (n = 8; Open bars). There was no difference in the protein expression of PHD-1 (D) and PHD-2 (E) in the lungs of the LBW lambs (n = 6; Closed bars) compared with the ABW lambs (n = 7; Open bars). Representative Western blot images from animals in each treatment group for PHD-1 (F) and PHD-2 (G) are presented.** MNE, mean normalised expression; ABW, Average birth weight; LBW, Low birth weight; *P<0.05 from ABW.

**Fig 6:**
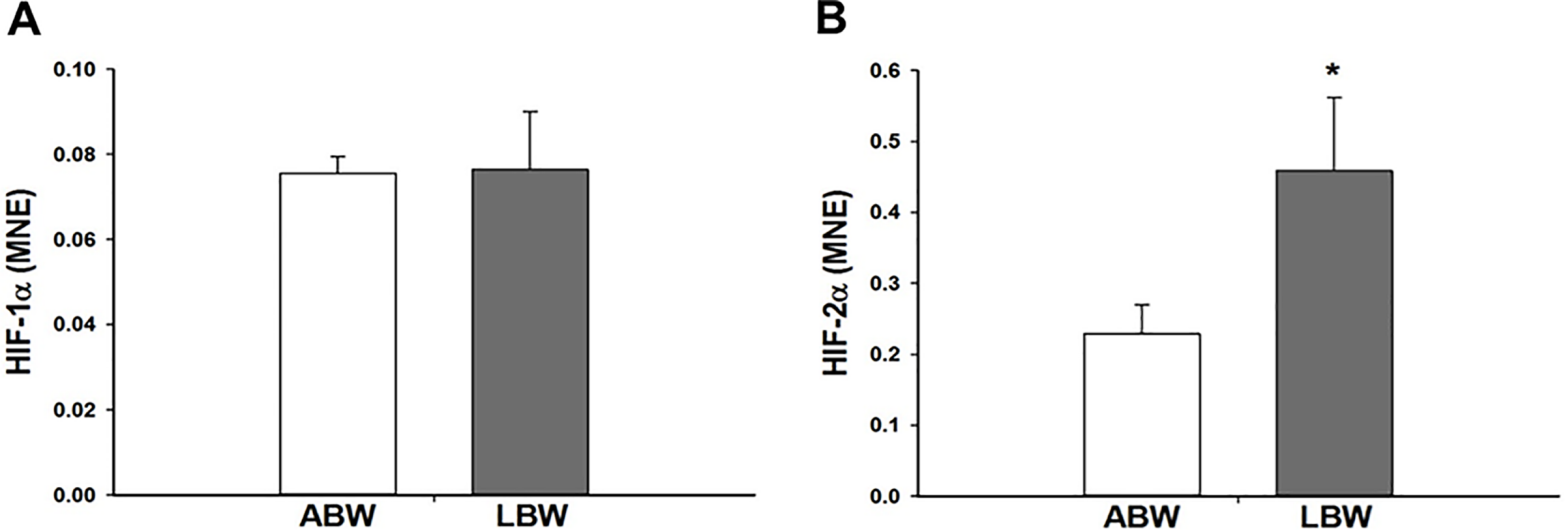
**There was no difference in the mRNA expression of *HIF-1α* (A) in the lungs of the LBW lambs (n = 6; Closed bars) compared with the ABW lambs (n = 9; Open bars). There was higher mRNA expression of *HIF-2α* (B) in the lungs of the LBW lambs (n = 6; Closed bars) compared with the ABW lambs (n = 8; Open bars).** MNE, mean normalised expression; ABW, Average birth weight; LBW, Low birth weight; *P<0.05 from ABW.

There was a decrease in *PHD-2* mRNA expression in the lung of LBW lambs (Fig 5B). However, there was no difference in *PHD-1* or *PHD-3* mRNA expression (Fig 5A and 5C) between the lungs of LBW and ABW lambs. There was also no difference in the protein expression of PHD-1 and PHD-2 in the lungs of LBW and ABW lambs (Fig 5D and 5E). There was an increase in *HIF-2α*, but not *HIF-1α* mRNA expression in the lungs of LBW lambs (Fig 6A and 6B). There was no effect of LBW on the mRNA expression of genes with hypoxia response elements (*VEGF*, *JMJD1A*, *IGF-2*, *FLT-1* and *ACE-1*; Table 3).

**Table 3:** mRNA expression of genes with hypoxia response elements in the lung of ABW and LBW lambs.

Gene Name (MNE)	ABW (n = 9)	LBW (n = 7)
*VEGF*	0.070 ± 0.004	0.084 ± 0.017
*JMJD1A*	0.026 ± 0.002	0.024 ± 0.003
*IGF-2*	1.147 ± 0.086	1.011 ± 0.145
*FLT-1*	0.081 ± 0.004	0.093 ± 0.015
*ACE-1*	0.044 ± 0.003	0.053 ± 0.008

Data (mean ± SEM) were analysed using Student’s unpaired t-test. MNE, mean normalised expression; ABW, average birth weight; LBW, low birth weight.

### Effect of LBW on mRNA expression of PPARγ and genes with PPARγ response elements in the lungs 21d after birth

There was no change in lung mRNA expression of *PPARγ* or *PAI-1*, a gene with a PPARγ response element, between ABW and LBW lambs (Table 4). However, there was higher *SPHK-1* mRNA expression, another gene that has a PPARγ response element, in the lungs of LBW compared with ABW lambs.

**Table 4:** mRNA expression of PPARγ and genes with PPARγ response elements in the lung of ABW and LBW lambs.

Gene name (MNE)	ABW (n = 9)	LBW (n = 7)
*PPARγ*	0.005 ± 0.0004	0.006 ± 0.0005
*SPHK-1*	0.00038 ± 0.00003	0.00049 ± 0.00004*
*PAI-1*	0.137 ± 0.02	0.142 ± 0.01

Data (mean ± SEM) were analysed using Student’s unpaired t-test. MNE, mean normalised expression; ABW, average birth weight; LBW, low birth weight

*P < 0.05.

### Effect of LBW on markers of proliferation, cell cycle regulators, markers of type II AECs and markers of pulmonary surfactant lipid synthesis in the lungs of lambs 21d after birth

There was no difference in the mRNA expression of markers of proliferation (*PCNA* and *KI-67*) or regulators of cell cycle progression (*p27* and *CCND-1*) in the lungs of LBW and ABW lambs (Table 5). However, LBW was associated with an increase in *FAS* mRNA expression, an enzyme involved in surfactant lipid synthesis, but there was no difference in the mRNA expression of *PCYT1A*, a rate limiting enzyme involved in surfactant phosphatidylcholine synthesis in the lung. LBW was associated with higher mRNA expression of *LAMP-3*, a marker of lamellar bodies present in type II AECs but there was no effect on surfactant lipid transporter *ABCA3* mRNA expression (Table 5).

**Table 5:** mRNA expression of markers of cellular proliferation and regulators of cell cycle progression, marker of lamellar bodies in type II AECs, pulmonary surfactant lipid synthesis and transport in the lung of ABW and LBW lambs.

Gene name (MNE)	ABW (n = 9)	LBW (n = 7)
**Proliferation and cell cycle progression**		
*PCNA*	0.015 ± 0.002	0.020 ± 0.006
*KI-67*	0.016 ± 0.001	0.024 ± 0.005
*p27*	0.027 ± 0.002	0.025 ± 0.002
*CCND-1*	0.017 ± 0.004	0.016 ± 0.002
**Marker of lamellar bodies in type II AECs**		
*LAMP-3*	0.088 ± 0.015	0.129 ± 0.024*
**Pulmonary surfactant lipid synthesis and transport**		
*PCYT1A*	0.041 ± 0.001	0.040 ±0.004
*FAS*	0.035 ± 0.001	0.045 ±0.002*
*ABCA3*	0.033 ± 0.002	0.037 ± 0.002

Data (mean ± SEM) were analysed using Student’s unpaired t-test. MNE, mean normalised expression; ABW, average birth weight; LBW, low birth weight

*P < 0.05.

## Discussion

In this study, we have shown that there is an increase in lung mRNA expression of *SP-A*, *-B* and *-C* in LBW compared with ABW lambs at 21 days after birth which is in contrast to the decrease in *SP-A*, *SP-B* and *SP-C* mRNA expression and protein abundance in the lung of the PR fetus in late gestation [27,28]. Despite the an absolute fold change of ~2.0-fold increase in *SP-A*and absolute fold change of ~1.7-fold increase in *-B* mRNA expression in the lung of LBW lambs in this study, there was no difference in the protein expression of either SP-A or -B. Hence, the deficit in lung tissue SP in the PR fetus observed in late gestation was normalised by 21d after birth in the LBW lamb, possibly as a result of the persistent elevation in SP mRNA expression. In this study, we have further examined the role of a range of intrapulmonary signalling pathways, including regulation by GCs, hypoxia, PPARγ, cellular proliferation and global methylation, which may be responsible for the altered regulation of surfactant maturation in the LBW lamb.

The dramatic increase in fetal plasma cortisol during late gestation in the normally grown fetus [55,56]coincides with important steps in lung structural and pulmonary surfactant maturation in preparation for postnatal life [57,58]. GCs increase the mRNA expression of *SP-A*, *-B* and *-C* [39,59,60]. In sheep, the PR fetus has higher plasma cortisol concentrations across late gestation compared with Control fetuses [27,61]. However, despite increased plasma cortisol concentrations in the PR fetus, there is reduced surfactant mRNA and protein expression in late gestation [27], which is associated with reduced GC signalling and altered hypoxia signalling [28]. As adult men born with LBW have higher plasma cortisol concentrations than men born ABW, we measured cortisol concentration and factors regulating glucocorticoid availability in the LBW lambs [62,63]. However, in the present study, we found that the higher plasma cortisol concentrations observed in the hypoxic PR fetus in late gestation were not sustained in the 21d old LBW lambs. In this study, we have measured the expression of regulators of cortisol availability; 11βHSD-1, which converts inactive cortisone to active cortisol, and 11βHSD-2, which converts active cortisol into inactive cortisone [64]. Despite an increase in the mRNA expression of *11βHSD-1*, we found no change in the 11βHSD-1 protein abundance in the lungs of the LBW lamb and this may be due to a small absolute fold change in mRNA expression of *11βHSD-1* of only ~1.18-fold Although there was an absolute fold change of ~1.3-fold increase in mRNA abundance of the *GR*, the intracellular mediator of GC action, this was not associated with an increase in the protein abundance of GR. Therefore, GC is unlikely to be responsible for the increased surfactant protein mRNA expression in the lung.

We found no change in the expression of key markers of cellular proliferation including *PCNA* and *KI-67* as well as genes involved in the regulation of cell cycle progression, *p27* and *CCND-1*, suggesting that there was no change in cell proliferation in lung tissue. In the present study we were unable to determine the numerical density of SP producing cells in the alveolar epithelium of lung tissue due to lack of fixed tissue availability and we therefore used *LAMP-3*, which is expressed in lamellar bodies, as a marker of type II AEC maturation. In normal developing sheep lungs, an increase in LAMP-3 correlates with a decrease in glycogen storage, a substrate for surfactant lipid synthesis, in type II AEC cells, which indicates cellular maturation [65]. In the present study, there was an increase in mRNA expression of *LAMP-3* (absolute fold change of ~1.73-fold) in the lungs of the LBW lambs, which may indicate either an increase in the number of type II AEC, or an increase in the number of lamellar bodies in the existing type II AEC, suggesting an increase in the maturity and synthetic capacity for surfactant production. Overall, the reasons for the lack of increase in the SP protein expression, despite an increase in *SP* mRNA expression, is unknown but may be due to either epigenetic changes in the lung or an increase in secretion of SP into the alveolar space. Moreover, to date neither we, nor to our knowledge, others have examined potential changes in the lipid component of surfactant in this lamb model of LBW following PR. A limitation of the current study is that we were unable to perform lung functional studies in these lambs or analyse surfactant composition in lung lavage. In 140d gestation fetuses, PR decreased total phosphatidylcholine content in lung lavage [41] while in a rat undernutrition model of IUGR, there was a decrease in lung tissue saturated phosphatidylcholine on postnatal day 1, which was normalised by 1 week of age [66]. Here we examined the mRNA expression of two key enzymes involved in the synthesis of pulmonary surfactant lipids, *PCYT1A* and *FAS* [67]. Interestingly, we found an increase in the mRNA expression of *FAS*, which may indicate that there is an increase in surfactant lipid synthesis in the lungs of the LBW lambs.

A further key regulator of surfactant development is the intracellular hypoxia signalling pathway, which is essential for normal lung development [68]. Although the fetus is hypoxemic during normal development relative to the newborn or adult [69], PR fetuses experience chronic hypoxemia compared with the normally grown fetus [16,70]. The hypoxia inducible factor (HIF)-α subunits mediate transcription of a range of genes involved in metabolism, angiogenesis, erythropoiesis, mitogenesis and apoptosis during periods of hypoxia [71]. Regulation of hypoxia signalling by the HIF-α subunit is negatively regulated by the prolyl hydroxylase domain (PHD) enzyme family during periods of normoxemia [71,72]. However, there is a change in this feedback mechanism during chronic hypoxemia resulting in increased PHD activity [73]. We have recently reported that PR increases both *PHD-2* and *PHD-3* in the lung of fetuses at 130 and 140d gestation [28] and increases in PHD-2 in the heart of 140d gestation fetuses [44]. These findings suggest that the exposure of the PR fetus to chronic hypoxemia *in utero* dynamically regulates hypoxia signalling in the lung in late gestation. In the present study, although there was no difference in the mRNA expression of *HIF-1α*, there an increase in absolute fold change of ~2-fold in *HIF2-α*.There was a small decreased in absolute fold change of ~0.75-fold in *PHD-2* mRNA expression in the lungs of LBW lambs and this may be the reason why the decrease is not reflected in the protein expression of PHD-2. Loss of HIF-2α in mice leads to decreased *SP-A*, *-B* and *-D* mRNA expression at birth, indicating the importance of HIF-2α in lung development and the production of SP [12]. Interestingly, the increase in *HIF-2α* but not *HIF-1α* mRNA expression was observed in LBW lambs and is in direct contrast to the observation in lungs of the late gestation PR fetus [28], but may explain the increase in SP mRNA expression in the lungs of LBW lambs. Interestingly, the changes observed in this study provide evidence for reduced feedback by PHDs to downregulate HIF-α expression in the postnatal normoxemic environment. This suggests that there may be a persistence of the upregulation of the hypoxia signalling pathway in the lung of the LBW lamb.

Activation of PPARγ signalling increases SP-B and -C abundance in the lung [9]. In addition, PPARγ deficient mice have changes in lung structure, including enlarged air spaces, increased lung volume and decreased tissue resistance [74]. In this study, we evaluated the mRNA expression of *PPARγ* and two genes regulated by PPARγ, namely *SPHK-1* [75] and *PAI-1* [76]. Despite no change in *PPAR*γ mRNA expression or its target *PAI-1*, we found an increase in *SPHK-1* mRNA expression in LBW lambs, which is unlikely to be PPARγ-mediated. *SPHK-1* expression can also be increased by HIF-2α [77], for which the mRNA was increased in the lungs of LBW lambs, providing further evidence for increased *HIF-2α* signalling.

In summary, although there is reduced surfactant maturation associated with altered regulation of factors important for lung maturation in the PR fetus in late gestation [27], surfactant maturation is normalised in the LBW lung by 21d after birth. It is unlikely that GC signalling is involved in regulating this postnatal surfactant maturation, as GC availability was not altered in the LBW lung. However, altered hypoxia signalling may be involved in the regulation of SP expression as we observed an increase in *HIF-2α* mRNA expression and decreased *PHD-2* mRNA expression in addition to an increase in *SPHK-1* mRNA expression, which may be driving this catch up in surfactant maturation observed in LBW lambs. *SPHK-1* mRNA expression can be regulated by both PPARγ and HIF-2α, and in this study, we have found no changes in the mRNA expression of *PPARγ* and *PAI-1*, suggesting that the increase in *SPHK-1* may be mediated by HIF-2α. Hence, our findings provide further evidence of altered molecular regulation, particularly associated with the hypoxia signalling pathway in postnatal life as a result of the compromised fetal development associated with IUGR.

## Abbreviations

11βHSD: 11β hydroxysteroid dehydrogenase
AEC: Alveolar epithelial cell
ACE: Angiotensin converting enzyme
ABCA3: ATP-binding cassette, sub-family A, member 3
ABW: Average birth weight
cDNA: Complementary deoxyribonucleic acid
PCYT1A: phosphate cytidylyl transferase 1, choline, alpha
CYCLO: Cyclophilin
FAS: Fatty acid synthase
FLT: Fms-related tyrosine kinase
GATA: GATA binding protein
GC: Glucocorticoid
GR: Glucocorticoid receptor
JMJD1A: Histone demethylase
HIF: Hypoxia inducible factor
IGF: Insulin-like growth factor
IUGR: Intrauterine growth restriction
LBW: Low birth weight
LAMP: Lysosome-associated membrane glycoprotein
MR: Mineralocorticoid receptor
PAI: Plasminogen activator inhibitor
PPARγA: Peroxisome proliferator-activated receptor gamma
PR: Placental restriction
PCNA: Proliferating cell nuclear antigen
PHD: Prolyl hydroxylase domain
RNA: Ribonucleic acid
RpP0: Ribosomal protein P0
SDS-PAGE: Sodium dodecyl sulfate polyacrylamide gel electrophoresis
SEM: Standard error of the mean
SPHK: sphingosine kinase
SP: Surfactant protein
TTF: Thyroid transcription factor
UPE: umbilicoplacental embolisation
VEGF: Vascular endothelial growth factor
ACTB: β actin
